# The development and use of decision support framework for informing selection of select agent toxins with modelling studies to inform permissible toxin amounts

**DOI:** 10.3389/fbioe.2022.1003127

**Published:** 2022-10-03

**Authors:** Segaran P. Pillai, Todd West, Rebecca Levinson, Julia A. Fruetel, Kevin Anderson, Donna Edwards, Stephen A. Morse

**Affiliations:** ^1^ Office of the Commissioner, Food and Drug Administration, United States Department of Health and Human Services, Silver Spring, MD, United States; ^2^ Sandia National Laboratories, U.S. Department of Energy (Retired), Livermore, CA, United States; ^3^ Sandia National Laboratories, U.S. Department of Energy, Livermore, CA, United States; ^4^ Science and Technology Directorate, U.S. Department of Homeland Security, Washington, D.C., WA, United States; ^5^ Centers for Disease Control and Prevention (Retired), Atlanta, GA, United States

**Keywords:** select agents and toxins, decision support framework, permissible toxin limits, public health impact, select toxins

## Abstract

Many countries have worked diligently to establish and implement policies and processes to regulate high consequence pathogens and toxins that could have a significant public health impact if misused. In the United States, the Antiterrorism and Effective Death Penalty Act of 1996 (*Public Law 104-132, 1996*), as amended by the Bioterrorism Preparedness and Response Act of 2002 (*Public Law 107-188, 2002*) requires that the Department of Health and Human Services (HHS) [through the Centers for Disease Control and Prevention (CDC)] establish a list of bacteria, viruses, and toxins that have the potential to pose a severe threat to public health and safety. Currently, this list is reviewed and updated on a biennial basis using input from subject matter experts (SMEs). We have developed decision support framework (DSF) approaches to facilitate selection of select toxins and, where toxicity data are known, conducted modelling studies to inform selection of toxin amounts that should be excluded from select agent regulations. Exclusion limits allow laboratories to possess toxins under an established limit to support their research or teaching activities without the requirement to register with the Federal Select Agent Program. Fact sheets capturing data from a previously vetted SME workshop convened by CDC, literature review and SME input were developed to assist in evaluating toxins using the DSF approach. The output of the DSF analysis agrees with the current select toxin designations, and no other toxins evaluated in this study were recommended for inclusion on the select agent and toxin list. To inform the selection of exclusion limits, attack scenarios were developed to estimate the amount of toxin needed to impact public health. Scenarios consisted of simulated aerosol releases of a toxin in high-population-density public facilities and the introduction of a toxin into a daily consumable product supply chain. Using published inhalation and ingestion median toxic dose (TD_50_) and median lethal dose (LD_50_) values, where available, a range of toxin amounts was examined to estimate the number of people exposed to these amounts in these scenarios. Based on data generated by these models, we proposed toxin exclusion values corresponding to levels below those that would trigger a significant public health response (i.e., amounts estimated to expose up to ten people by inhalation or one hundred people by ingestion to LD_50_ or TD_50_ levels of toxin in the modeled scenarios).

## Introduction

The use of biological agents and toxins for nefarious purposes (i.e., bioterrorism, biological warfare, and bio-criminality) and efforts employed to thwart their use are of utmost importance to the public health and law enforcement communities. For the purposes of this paper, we are defining bioterrorism as the threat or use of biological agents and toxins (i.e., viruses, bacteria, fungi, or their toxins) by individuals or groups motivated by political, religious, ecological, or other ideological objectives, while biological warfare (BW) is defined as a specialized type of warfare using biological agents or toxins conducted by a government against a target (Ryan and Glarum, 2008). We do not consider the use of biological toxins for entirely nonpolitical goals (such as revenge or profit) as bioterrorism, but rather bio-criminality. This paper focuses on toxins, which are harmful substances produced by plants or within living cells or organisms. Toxins can be small molecules, peptides, or proteins that are capable of causing illness after contact with, or absorption by body tissues interacting with biological macromolecules such as enzymes or cellular receptors. Toxins have been used for nefarious purposes by bioterrorists and criminals, as well as produced and stockpiled for use in BW programs.

The earliest recorded use of toxins in warfare was in the sixth century B.C. when Assyrians contaminated the water supply of their enemies with the fungus *Claviceps purpurea* (rye ergot), which produces alkaloids related to lysergic acid (Haarmann et al., 2009). The first known examples of state-supported bioterrorism with a toxin occurred in 1978 and involved the Bulgarian Secret Police’s attempted assassination of Vladimir Kostov, a Bulgarian defector who had served as a news correspondent and was also a major in the D. S. (Bulgarian equivalent of the K. G. B.). A small metal pellet was injected into Kostov who subsequently became ill but did not die (Rozsa and Nixdorff, 2006). In a separate incident the same year in London, the Bulgarian Secret Police assassinated Georgi Markov, a dissident and announcer for Radio Free Europe, using technology supplied by the Soviet Union (Rozsa and Nixdorff, 2006). He died 4 days after a small platinum-iridium pellet containing ricin was injected into the back of his thigh by means of a modified umbrella tip.

In 1991, members of the Minnesota Patriots Council attempted to poison an Internal Revenue Service (IRS) official, local law enforcement, and a U. S. Deputy Marshal with ricin that they had extracted from castor beans purchased from a mail order house. They planned to use dimethyl sulfoxide to deliver the ricin transdermally. Four members were arrested after the group was infiltrated by the Federal Bureau of Investigation (FBI) (Carus, 2002).

Between 1990 and 1995, the Aum Shinrikyo, a doomsday religious cult, attempted on at least 10 occasions to disperse biological agents and toxins against the civilian population and authority figures in Japan (Wheelis and Segistima, 2006). Three of these incidents involved the dispersal of a crude preparation of botulinum toxin. In 1990, they outfitted a car to disperse liquid material through the exhaust system and drove the car around the parliament building. In 1993, they attempted to disrupt the wedding of Prince Naruhito by spreading botulinum toxin in downtown Tokyo *via* an automobile. In 1995, they planted three briefcases designed to release botulinum toxin in a Tokyo subway. This attack was unsuccessful because a cult member substituted a non-toxic agent for the toxin (Carus, 2002).

Toxins have also been used by individuals for nefarious purposes. For example, in 1995, Dr. Debora Green, an oncologist, attempted on three occasions to kill her estranged husband (Dr. Michael Farrar) by putting ricin in his food (Carus, 2002). A more recent demonstration of the continuing threat from ricin occurred in 2003 when a letter signed “Fallen Angel” complaining about new federal trucking regulations and a threat to use ricin was discovered in a postal facility in Greenville, South Carolina. The letter was enclosed in a package with a vial containing ricin and addressed to the White House (Schier et al., 2007). Numerous incidents and hoaxes involving ricin have occurred in the United States (Department of Health and Human Services, 1996; Carus, 2002). In 2015, Jesse William Korff, a 20-year-old Florida man was sentenced to 110 months in prison after he was found guilty of producing, transferring, selling, and smuggling abrin and ricin, and conspiring to kill a woman in the United Kingdom. Korff had advertised the sale of these toxins on the dark web and provided prospective purchasers with information about the quantities necessary to kill a person of a given weight, along with instructions how to secretly administer the toxin to avoid suspicion by law enforcement officers (Department of Justice Office of Public Affairs, 2015).

Toxins (both lethal and incapacitating) have been part of state-supported BW programs including those of Canada, France, the U.S., U.S.S.R., U.K., South Africa, Iraq, Syria, Japan, and North Korea (Chemical and Biological Weapons: Possession and Programs Past and Present.pdf, 2021). Most of these programs were discontinued and stocks of agents destroyed after the Biological Weapons Convention came into force in 1975. However, not all countries complied with the agreement. In 1995, Iraqi authorities acknowledged they had loaded toxins into various types of munitions. These included 100 botulinum toxin bombs, 16 aflatoxin bombs, 13 botulinum toxin and 2 aflatoxin missile warheads, and 122-mm rockets filled with botulinum toxin and aflatoxin (Mangold and Goldberg, 1999).

These events and others have led to changes in the way scientists in the United States and other countries acquire and work with certain toxins. For example, more than 20 years ago the U.S. promulgated regulations (i.e., Select Agent Regulations, 2022) designed to ensure the biosafety and biosecurity of activities involving the possession and use of hazardous biological select agents and toxins, and the facilities in which these activities occur (Morse, 2015). A key component of these regulations is a list of biological agents and toxins (i.e., select agents and toxins) that have the potential to pose a severe threat to human health and safety and economically impact agriculture through threats to animal and plant health or their products. CDC was delegated by HHS to administer the Select Agent Regulations that pertain to human health. The Bioterrorism Preparedness and Response Act of 2002 (*Public Law 107-188, 2002*) requires that the list of select agents and toxins be reviewed and republished at least biennially and that the HHS Secretary consider specific criteria in determining whether to include a biological agent or toxin on the list. With respect to toxins these criteria are: 1) the effect on human health from exposure to the toxin; 2) the methods by which the toxin is transferred to humans; 3) the availability and effectiveness of pharmacotherapies and immunizations to treat and prevent illness resulting from a toxin; and 4) any other criteria including the needs of children and other vulnerable populations that the HHS Secretary deems relevant. The Select Agent Regulations outlines an exclusion to the regulations as long as the aggregate amount of the toxin (e.g., the aggregrate amount of serotype A–G for botulinum toxin or A–E for staphylococcal enterotoxins) under the control of a principal investigator (PI), treating physician or veterinarian, or commercial manufacturer or distributor does not, at any time, exceed the permissible limit.

We evaluated risk-based multi-criteria decision analysis techniques to inform select agent designation, applying the approach broadly to include non-select agents to evaluate its generality (Pillai et al., 2022). Toxins were not included in this previous evaluation since identifying toxins for inclusion on the list of select toxins and assigning exclusion limits presented some unique challenges including: 1) toxins are non-replicating; 2) toxins are not transmissible from person-to-person; 3) while some are toxic at very low doses (e.g., botulinum toxin), they are often produced or present in small amounts requiring purification and concentration; 4) some active toxins may have medical uses (e.g., botulinum toxin); 5) some toxins are produced by microorganisms commonly encountered in the clinical laboratory (e.g., staphylococcal enterotoxins); and 6) some toxins are waste products of industrial processes (e.g., ricin).

We have developed and applied DSF approaches for addressing these challenges and for assessing toxins for inclusion as select toxins. In addition, we developed attack scenarios to determine the amount of toxin needed to impact public health and applied the results to suggest exclusion limits.

The attack scenarios involved the introduction of toxin into a fluid daily consumable product supply chain to simulate an ingestion scenario and the release of an aerosolized toxin into three high-population density public facilities to simulate inhalation scenarios. By design, these scenarios were meant to capture close to “worst case” conditions from the defender’s perspective. Real-World attempts would likely produce fewer exposures than modeled in this study. Excluded toxin limits (i.e., amount of toxin under an established limit to support a PI’s research or teaching activities without the requirement to register with the Federal Select Agent Program) were based on the amount estimated to expose up to 10 people by inhalation or up to 100 people by ingestion to median LD_50_ or median TD_50_ of toxin. These thresholds were chosen based on SME input for the number of casualties that would be considered a significant public health threat and/or associated response. It is likely that the number of people exposed to amounts below the LD_50_ or TD_50_ will be much greater for these scenarios, and the number of people who believe themselves to be exposed, should the release be made public, may be greater yet.

A key limitation to the modelling approach has been the lack of LD_50_ or TD_50_ data for inhalation and ingestion routes of exposure for some toxins of potential interest. In this study, all the toxins modeled to inform recommendation of permissible levels have at least one known or published LD_50_ or TD_50_ value. An alternative approach was also explored that solicited SME input regarding the amount of toxin considered reasonable for a PI to possess for conducting laboratory research or teaching. The overall intent of this study was to present a methodology using the DSF and associated exposure scenario results in sufficient detail so that other countries and responsible entities can employ these methods as additional data become available, thus continuously improving the process.

## Materials and methods

### Toxins

Toxins were chosen for this analysis based on their current or former inclusion as an HHS select toxin or those on other countries regulated lists, previous efforts by state chemical and/or biological weapons programs to weaponize a particular toxin or were otherwise toxins of public health interest (Table 1). To evaluate the toxins using the DSF approach, we developed toxin fact sheets using data from a previously vetted SME workshop convened by CDC (unpublished data), literature reviews, and SME input. These data included: etiological agent, ease of production, time to onset of symptoms, LD_50,_ TD_50,_ mortality rate, stability in various matrices (water, food, and air), number of known human cases, human exposure routes, clinical syndromes and/or symptoms, therapy, and prophylaxis.

**TABLE 1:** Toxins included in this analysis. Toxins were chosen based on various countries’ Select Agent Lists, inclusion in previous offensive chemical and/or biological weapons programs (Anti-terrorism, Crime and Security Act 2001, 2001; Security Sensitive Biological Agents, 2020; Security Sensitive Biological Agents and Toxins, 2021; Infectious agents and toxins, 2007; Chemical and Biological Weapons: Possession and Programs Past and Present.pdf, 2008; Risk Group Database, 2021; and Biological and chemical threats, 2014) or were otherwise of public health interest.

Toxin	Rationale for inclusion in this study^a^
Botulinum^b^	**Select Agent Toxin list:** US, Canada, UK, S. Korea, Australia, Japan, EU
	**Former BW program toxin:** Canada, France, Iran, Iraq, N. Korea, S. Africa, Syria, UK, US
Abrin	**Select Agent Toxin list:** US, UK, Australia, EU
Ricin^b^	**Select Agent Toxin list:** US, UK, Australia, EU
	**Former BW program toxin:** Canada, France, Iraq, S. Africa, Syria, US
Staphylococcal enterotoxin B	**Select Agent Toxin list:** US, Canada, UK, EU
	**Former BW program toxin:** US
Staphylococcal enterotoxins A, C, D, E	**Select Agent Toxin list:** US, UK
Conotoxins	**Select Agent Toxin list:** US, UK, EU
Saxitoxin	**Select Agent Toxin list:** US, UK, EU
	**Former BW program toxin:** US
Tetrodotoxin^b^	**Select Agent Toxin list:** US, UK, EU
	**Former BW program toxin:** Japan
Diacetoxyscirpenol toxin	**Select Agent Toxin list:** US, EU
T-2 toxin	**Select Agent Toxin list:** US, EU
Shiga and Shiga-like toxin	**Select Agent Toxin list:** Canada, UK, Japan, EU
*Clostridium perfringens* Epsilon toxin	**Select Agent Toxin list:** UK, EU
*Viscum album* Lectin 1 (Viscumin)	**Select Agent Toxin list:** UK, EU
Volkensin toxin	**Select Agent Toxin list:** UK, EU
*C. perfringens* enterotoxins	**Select Agent Toxin list:** UK, EU
Aflatoxins	**Select Agent Toxin list:** EU
	**Former BW program toxin:** Iraq
Alpha toxin	**Select Agent Toxin list:** Canada
Cholera toxin^b^	**Select Agent Toxin list:** EU
	**Former BW program toxin:** France, Japan, N. Korea, S. Africa
Cyanginosin (Microcystin)	**Select Agent Toxin list:** EU
Staphylococcal alpha-hemolysin	**Select Agent Toxin list:** Canada
Modeccin	**Select Agent Toxin list:** EU
Tetanus toxin	**Former BW program toxin:** Japan
Diphtheria toxin^b^	Public health interest
Pertussis toxin	Public health interest
Fumonisin toxin	Public health interest
Cylindrospermopsin toxin	Public health interest
Alpha-Amanitin	Public health interest

a US, United States; UK, United Kingdom; S. Korea, South Korea; N. Korea, North Korea; S. Africa, South Africa; EU, European Union.

b Previous terrorist interest or use as noted in Carus (2002).

### Decision support framework

The DSF approach applies key criteria using a logic tree format to identify those toxins which may be of sufficiently low concern that they can be ruled out from further consideration as a select toxin. While it is difficult to predict what toxins a terrorist might employ, the DSF, in accordance with the Bioterrorism Preparedness and Response Act of 2002, aims to identify those toxins that have the potential to pose a severe threat to public health and therefore would be most concerning, would terrorists be able to obtain them.

**FIGURE 1:**
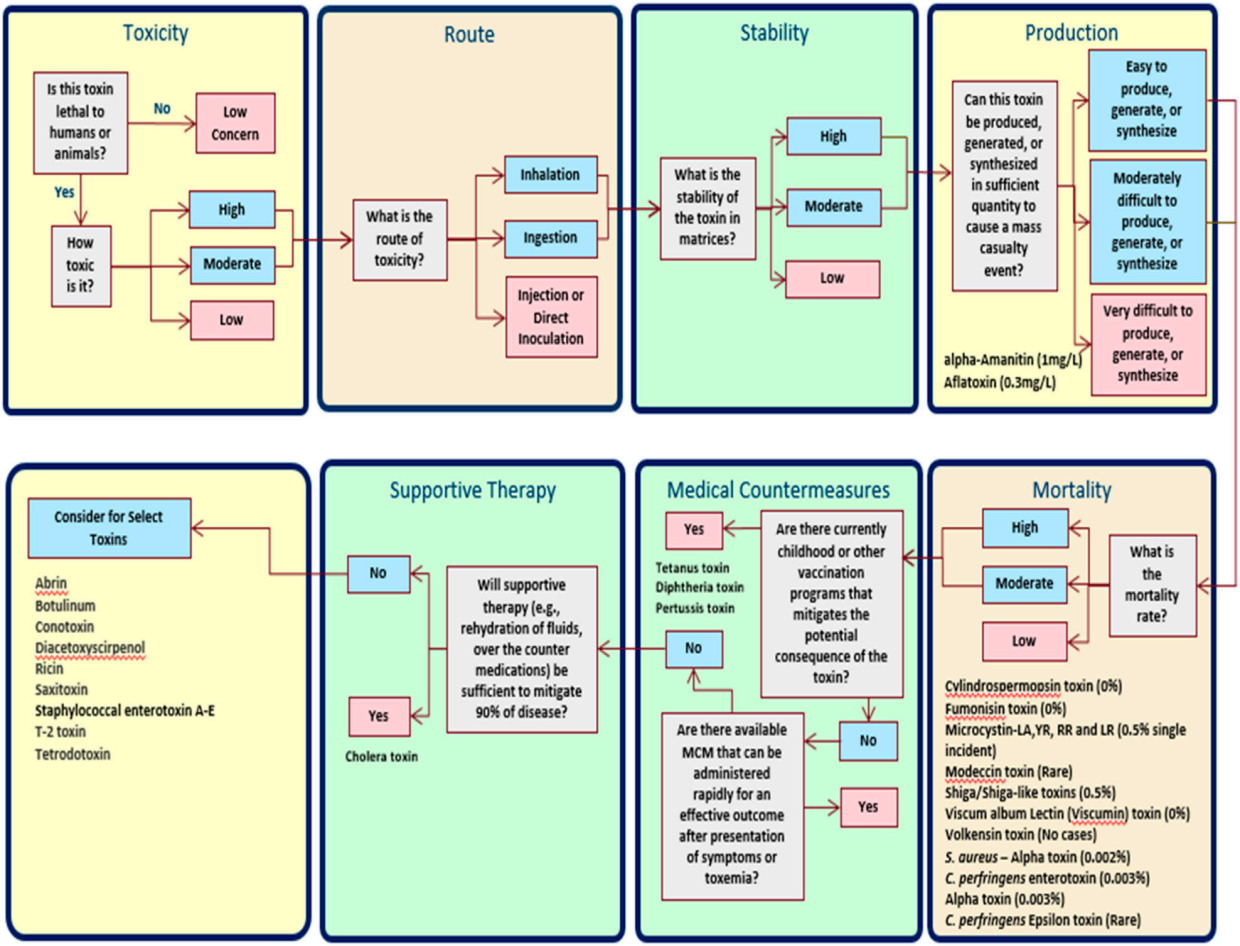
Assignments of Select and non-Select Toxins using DSF. If the result of any of the modules shows that a candidate toxin is of low risk with respect to that specific parameter, the toxin is removed from consideration, the output is flagged by a pink box, and there is no outgoing arrow—the analysis stops. If the analysis shows that the toxin is of high or moderate risk, the corresponding output box is blue, and an arrow continues to the next stage.

The DSF logic tree shown schematically in Figure 1 addresses the following questions: 1) is the toxin lethal to humans or animals, and if so, how toxic is it; 2) what is the potential route for exposure and toxicity; 3) what is the stability of the toxin in food, environment, or other matrices; 4) can the toxin be produced or synthesized in sufficient quantities to cause a mass casualty event; 5) what is the mortality rate; 6) are there currently childhood or other vaccination programs that mitigate the potential consequence of the toxin, 7) are there medical countermeasures that can be administered rapidly for an effective outcome after presentation with symptoms of toxemia; and 8) will supportive therapy (e.g., rehydration, over the counter medications) be sufficient to mitigate ≥90% of toxicity/disease.

Using the DSF, a toxin that does not meet a threshold value for at least one of the set criteria is deemed to be of Low Concern and thus is not considered as a select toxin for secondary evaluation to determine exclusion limits. Those toxins that exceed all criteria thresholds are considered for inclusion as a select toxin. If corresponding LD_50_ or TD_50_ values were available, those toxins were further evaluated using inhalation and/or ingestion attack scenarios (see below).

### Scoring

The broad criteria in Figure 1 were scored using definitions for High, Moderate, and Low Concern as listed in Table 2.

**TABLE 2:** Scoring definitions for assignment of High, Moderate or Low Concern using the DSF.

Criteria	High concern	Moderate concern	Low Concern
Toxicity	LD_50_ or TD_50_ < 50 µg/kg	LD_50_ or TD_50_ = 50–1,000 µg/kg	LD_50_ or TD_50_ > 1,000 µg/kg
Route	Inhalation and ingestion	Inhalation or ingestion	Injection/cutaneous
Stability	Highly stable in relevant environment and food matrices	Moderately stable in relevant environment and food matrices	Unstable in relevant environment and food matrices
Production	Easy to produce, generate or synthesize; e.g., isolation from culture supernatant or synthesis in high yields	Moderately difficult to produce, generate or synthesize; e.g, isolation from culture supernatant or synthesis in low yields	Very difficult to produce, generate or synthesize; e.g., difficult to culture or synthesize, low yields, slow grower
Mortality	>10%	1%–9%	<1%
Availability of MCM	No	No	Yes
Availability of childhood vaccination program	No	No	Yes
Efficacy of supportive therapy and/or over the counter medications	No	No	Yes

### Attack scenarios

Ingestion and inhalation attack scenarios were developed to assess the amount of toxin necessary to expose a defined population to LD_50_ or TD_50_ amounts. By design, these scenarios were meant to capture “worst case” conditions from the defender’s perspective; real-world attempts would likely produce fewer exposures than modeled in this study.

### Ingestion scenario modeling

The ingestion scenario consisted of the introduction of 1 mg to 1 kg amounts of toxin into the daily consumable supply chain of Product X. The identity of Product X is not provided due to the potential to reveal attack vulnerabilities. The details of toxin production and methods of introduction were not considered. The scenario assumes the product contained the specified quantity of active toxin upon reaching store shelves. Therefore, if processing would inactivate the toxin, the assumption was made that the toxin was introduced post-processing.

The total volume of contaminated product depended on the mass of toxin assumed to be available (varies from 1 mg to 1 kg) and the toxin concentration chosen by the attacker as illustrated in Eq. 1:

$$\text{Total volume of contaminated product =}\frac{\mathrm{mass of toxin available}}{\mathrm{toxin concentration}}$$ (1)

The final total volumes of contaminated Product X ranged from less than 1 L–10^8^ L. The largest scenarios, which involve contaminating tens of millions of servings, may not be possible in practice.

The scenario assumes contaminated Product X was purchased over a 2-day period and consumed over a 6-day period at a uniform rate. Consumption of Product X was assumed to follow age-dependent patterns (U.S. Department of Agriculture, 2004) until the contamination was recognized, a product recall, and an alert to stop consumption was issued. In keeping with the worst-case assumptions chosen for this analysis, it was assumed that all contaminated Product X was consumed before the recall and health advisory to consumers to cease consuming the product could be issued. For toxins other than saxitoxin and tetrodotoxin, the attacker chose a toxin concentration such that a person in the 45+ age group would consume 1 LD_50_ (or TD_50_) over the 6-day consumption period (consumption rate of 3.0 ml/mg/day). For example, for a 1,000 mg attack with a toxin that has an LD_50_ of 1 mg/kg, the volume of contaminated product would be as illustrated in Eq. 2:

$$\frac{1000\;mg*\;3.0\frac{ml}{kg\;day}\;*\;6\;days}{1\frac{mg}{kg}\;*\;1000\;mL/L}=18\;L$$ (2)

For a 1,000,000 mg (1 kg) attack with the same toxin, the volume of contaminated product would be 18,000 L. Since saxitoxin and tetrodotoxin are largely excreted within 24 h of consumption (Yu et al., 2010; DeGrasse et al., 2014), the scenario assumed the attacker would choose a toxin concentration such that a person in the 45+ age group would consume 1 LD_50_ (or TD_50_) over a 24-h period. If the toxin LD_50_ or TD_50_ in the literature was given by a range, then the geometric mean of this range was used.

### Inhalation scenario modeling

**FIGURE 2:**
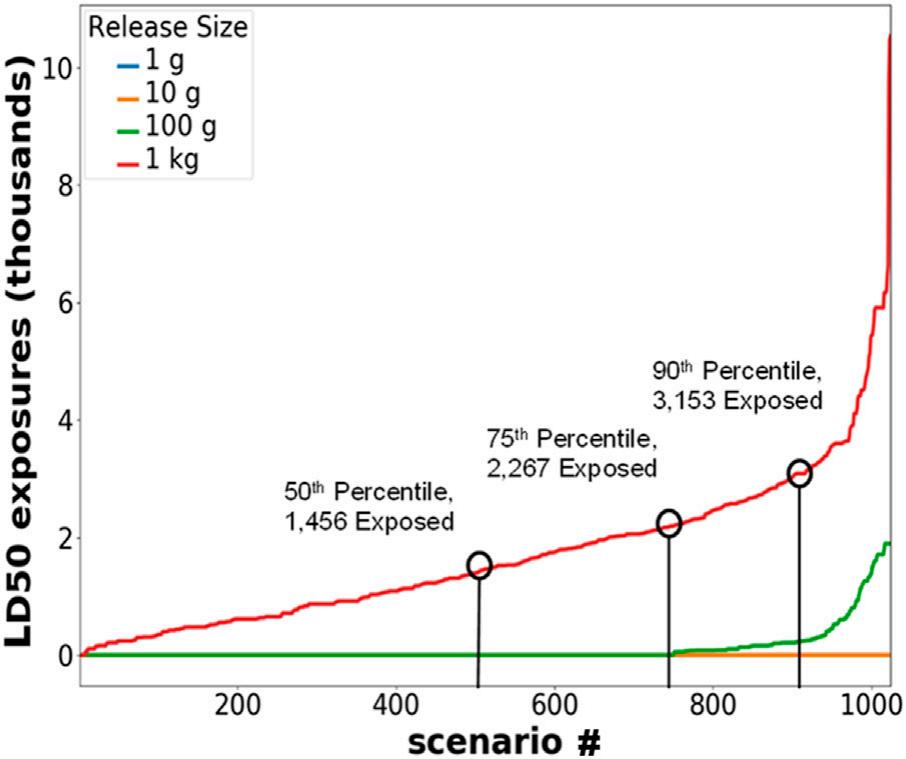
Example percentile calculation. Abrin LD_50_ exposures for 1,024 simulated releases in Facility 1, ordered from least to greatest number of LD_50_ exposures.

Three public facilities with high population densities were chosen for evaluation of toxin inhalation scenarios. The identity of these facilities is not included in this paper due to the potential vulnerabilities. Indoor releases were chosen over outdoor releases since releases into the confined space of a facility require smaller amounts of toxin to generate sufficient exposure than for outdoor releases. The three considered facilities have diverse layouts, from relatively segmented to a large open space, providing correspondingly diverse scenario outcomes. For each simulation, it was assumed that potential release locations and total population were evenly spaced throughout the occupied area. To evaluate potential aerosol movements and population exposures, facility airflow models previously developed and validated were used (U. S. Detecting Bioterrorist Attacks, 2021). These models used the National Institute of Standards and Technology (NIST) multizone CONTAM modeling software (Dols et al., 2015) and are based on data from detailed architectural drawings, site visits, and on-site measurements. These models were validated using simulant release in one of the facilities. For each facility, 1,024 simulated release scenarios were generated for each toxin. The scenarios consisted of 32 random release locations each for 32 random release times, with particle sizes ranging from 1–10 µm. It was assumed that no immediate symptoms occurred, and the release was not immediately detected, so there would be no changes in population movement during the release. All people were assumed to have the same mass of 70 kg, so the analysis did not consider the impacts of lower doses on children. A respiration rate of 10 L/min was assumed for each person. The amount of toxin released increased along a logarithmic scale from 1 mg to 1 kg. Since each simulated release in a facility was at a different location or time, each scenario run resulted in a different number of people who were theoretically exposed to LD_50_ and/or TD_50_ toxin levels. The results for each scenario were reported for the 50th (median), 75th, and 90th percentile exposure levels. An example is shown in Figure 2. Full results are available from the lead author by request. In keeping with the worst-case scenario assumptions in this analysis, 90th percentile exposure levels (i.e., number of exposures that is exceeded in only 10% of scenarios for a given toxin release amount) were used to determine permissible toxin amounts.

## Results

A DSF was developed and used to evaluate 30 toxins (Table 1) for consideration and inclusion as a select toxin. To assist in the evaluation of toxins against the DSF criteria, we developed fact sheets based on literature reviews coupled with SME input and review. These data are summarized in Table 3. At each decision point in the framework, toxins may drop from further consideration as a possible select toxin. For example, the “Production” criterion (Figure 1) resulted in the elimination of alpha-Amanitin and Aflatoxin from further consideration since they were scored as very difficult to produce, generate, or synthesize in sufficient quantity to cause a mass casualty event by a non-state actor. Another 11 toxins are eliminated from consideration as a select toxin because of low mortality rates (<1%), three toxins are eliminated due to existence of effective vaccination programs, and one toxin (cholera toxin) is eliminated due to the effectiveness of supportive therapy at mitigating ≥90% of disease. Botulinum toxin, while having an effective medical countermeasure for treatment, is not eliminated by the DSF due to the severity of illness, the long duration for recovery, the lack of rapid availability of medical countermeasures and the need for intubation and mechanical ventilation thus limiting countermeasures’ effectiveness in a bioterrorism attack resulting in mass exposures (Rao et al., 2021). In the case of staphylococcal enterotoxin (A–E), although the mortality rate is low in natural clinical cases, the environmental stability, resistance to high temperature, high morbidity rate (serving as incapacitating weapon) and potential mortality rate in an aerosol bioterrorism scenario was taken into consideration in the DSF (Hale, 2012).

**TABLE 3:** Summary of data used to support DSF analysis. Toxicity, route, stability, production, mortality, and availability of medical countermeasures data for 30 evaluated toxins (See Supplemental Reference List for references).

Toxin	Toxicity: LD_50_ or TD_50_ (µg/kg)			Route	Type of molecule	Production (source, yield)	Mortality	MCM
	Inhalation	Ingestion	Injection					
Select agents								
Abrin	3.3	100	2	All 3	Protein	Plant	5%	No
Botulinum toxin	0.01	1	0.0013–0.0024	All 3	Protein	Bacteria, 0.6 mg/L	5%–10% (treated); ≤60%	No^1^
Conotoxin	>18–22	Unknown	10–100	Inhalation, cutaneous	Peptide	Peptide synthesis	70%	No
Diacetoxyscirpenol	Unknown	7,000	380–12,000	All 3	Small molecule	Fungus, 550 mg/L	60% (mycotoxins)	No
Ricin	3–10	20,000–100,000	0.5–4	All 3	Protein	Plant	∼6%	No
Saxitoxin	2	300–1,000	3.4 (i.v.)	All 3	Small molecule	Cyanobacteria	15%	No
Staphylococcal enterotoxin A	Unknown	1	11–1,600	All 3	Protein	Bacteria, ∼100 mg/L	1.54%	No
Staphylococcal enterotoxin B	0.2	40–700	11–1,600	All 3	Protein	Bacteria, ∼200 mg/L	1.54%	No
Staphylococcal enterotoxin C	Unknown	3.3 (ED)	11–1,600	All 3	Protein	Bacteria, ∼400 mg/L	1.54%	No
Staphylococcal enterotoxin D	Unknown	5–10 (ED)	11–1,600	All 3	Protein	Bacteria, !1 mg/L	1.54%	No
Staphylococcal enterotoxin E	Unknown	3–10 (ED)	11–1,600	All 3	Protein	Bacteria, ∼30 mg/L	1.54%	No
T-2 toxin	50–2,000	1,800–10,500	500–5,200	All 3	Small molecule	Fungus, 500 mg/L	≤60%	No
Tetrodotoxin	2	15–60	8–14	All 3	Small molecule	Fish, 9 g/kg; lengthy synthesis	11%–15%	No
Non-select agents								
Aflatoxin B_1_	Unknown	300–18,000	Unknown	All 3	Small molecule	Fungus, 0.3 mg/L	27%–60% (*Aspergillus*)	No
Alpha toxins	Unknown	Unknown	3–5	Ingestion, cutaneous	Protein	Bacteria, 1–3 mg/L	∼0.003% (*C. perfringens*)	No
alpha-Amanitin	Unknown	100	Unknown	All 3	Small molecule	Mushrooms, 1 mg/L. slow grower	5%–30%	No
Cholera toxin	Unknown	250	Unknown	Ingestion	Protein	Bacteria, 20 mg/L	1-3% (*V. cholerae*)	Yes
*Clostridium perfringens* enterotoxins (CPEs)	Unknown	Unknown	81	Ingestion	Protein	Bacteria, 15 mg/L	∼0.003% (*C. perfringens*)	No
Cyanginosin: Microcystin-LR	43	5,000	25–150 (i.p.)	All 3	Small molecule	Cyanobacteria, 300 mg/kg	65% (single incident)	No
Cylindrospermopsin toxin	Unknown	4,400–6,900	64,000	Ingestion	Small molecule	Cyanobacteria, 5 mg/L	∼0% (*C. raciborskii*)	No
Epsilon toxin	Unknown	Unknown	0.07–0.11 (activated with trypsin)	Ingestion, cutaneous	Protein	Bacteria; yields not found	Unknown. ≤100% in sheep (*C. perfringens* types B, D)	No
Fumonisin toxin	Unknown	Unknown	Unknown	Ingestion	Small molecule	Fungus, 500 mg/L	∼0% (single outbreak)	No
Modeccin toxins	Unknown	Unknown	0.9–5.3	Ingestion	Protein	Plant, 200-2000 mg/kg	Rare (*A. digitata*)	No
Shiga- and shiga-like toxins	3	145	50	All 3	Protein	Bacteria, 0.1 mg/L	0.5% (*E. coli* 0157:H7)	No
*Staphylococcus aureus*—Alpha toxin	Unknown	Unknown	0.04–0.06	Ingestion	Protein	Bacteria, amounts not found	0.002% (*S. aureus*)	No
Tetanus toxin	Unknown	Unknown	0.0025 (wound)	Cutaneous	Protein	Bacteria, 200 mg/L	11% (*C. tetani*)	No
Viscum album Lectin ^1^ (Viscumin) toxins	Unknown	Unknown	2.1–80	Ingestion	Protein	Plant, 80–400 mg/kg	∼0% (*V. album*)	No
Volkensin toxin	Unknown	Unknown	1.4	Ingestion	Protein	Plant, 400–750 mg/kg	No cases reported (*A. volkensii*)	No
Diphtheria toxin	Unknown	Unknown	<0.1	Inhalation, cutaneous	Protein	Bacteria	5%–10%	Yes
Pertussis toxin	Unknown	Unknown	18	All 3	Protein	Bacteria	0.8%–6.5%	Yes

Although antitoxin for Botulinum toxin is available, treatment requires IV administration, on-going monitoring, and typically extended hospital stays even with antitoxin; therefore, it is scored as ‘No’ under MCM column.

The results of this DSF analysis indicate all currently designated HHS select toxins, and none of the other toxins considered in Table 1, are recommended for consideration as a select toxin (Figure 1).

Inhalation and ingestion scenarios were modeled for those toxins recommended for consideration as a select toxin and where the inhalation or ingestion LD_50_ or TD_50_ values are known (all recommended toxins have at least one known LD_50_ or TD_50_ value). Table 4 summarizes toxin release amounts needed to generate exposure to an LD_50_ or TD_50_ for at least 10 people in analyzed inhalation scenarios and at least 100 people in analyzed ingestion scenarios, as calculated from our models. Based on these results, the proposed toxin permissible limit is suggested to be the lesser of the results from the inhalation and ingestion scenarios (Table 4).

**TABLE 4:** Summary of toxin release amounts needed to generate exposure to an LD_50_ or TD_50_ for at least 10 people in analyzed inhalation scenarios and at least 100 people in analyzed ingestion scenarios, as calculated by our models*; SME recommended levels for not impeding laboratory research; and current Select Toxins Permissible Limits as of June 2022.

Toxin	Inhalation scenario (≤10 cases) (mg)*	Ingestion scenario (≤100 cases) (mg)*	Lesser of columns 2 and 3 (mg)	SME recommended research levels (mg)**	Current select agents and toxins program permissible limit (mg)^2^
Abrin	10,000	10,000	10,000	1,000	1,000
Botulinum toxin	50	10	10	10	1
Conotoxin	100,000	N/A	100,000	1,000	100
Diacetoxyscirpenol	N/A	100,000	100,000	1,000	10,000
Ricin	10,000	100,000	10,000	1,000	1,000
Saxitoxin	10,000	1,000	1,000	1,000	500
*Staphylococcol* enterotoxin B	1,000	1,000	1,000	1,000	100
T-2 toxin	1,000,000	10,000	10,000	1,000	10,000
Tetrodotoxin	10,000	1,000	1,000	1,000	500

* Appropriate entities with a need to know can reach out to the lead author for this information. ** This is not a statutory criterion used to evaluate select toxins.

An alternative approach was also explored that solicited SME input regarding the maximum amount of toxin considered reasonable to possess for conducting laboratory research or teaching. A summary of the SME recommendations regarding reasonable levels for laboratory research are provided in Table 4. Other than botulinum neurotoxin, the SMEs felt that most scientific research can be performed effectively with 1 g of material, and as such there is no need for any non-select agent registered laboratories to possess greater than 1 g of purified toxin material at any given time, suggesting 1 g for consideration as an exclusion limit. For botulinum toxin, the SMEs felt a total of 10 mg of purified toxin from all serotypes is the reasonable limit to possess for conducting research or teaching purposes in non-select agent registered laboratories.

## Discussion

The goal of this analysis was to inform selection of toxins for inclusion as a select toxin and associated exclusion limits. The original rationale for these limits was based on primary and secondary criteria. Primary criteria focused on the potential of the toxin as an aerosol threat. Secondary criteria focused on a variety of scenarios and historical use, including the potential of the toxin as an ingestion threat with a focus on food safety and/or typical quantities of toxin commonly used in biomedical research. The DSF and ingestion and inhalation scenario results described in this report support these primary and secondary criteria. While the DSF does not rule out any of the considered toxins because of their toxicity, it does rule out toxins as a select toxin based on factors that would play into the likelihood and consequences of a bioterrorism scenario, such as difficulty to produce a sufficient quantity to cause a mass casualty event, existence of effective vaccines and therapies, and overall mortality rate.

Comparison of proposed exclusion levels for select toxins based on the inhalation and ingestion modeling studies with the SME guidance on maximum research amounts (Table 4) shows agreement for botulinum toxin, saxitoxin, *Staphylococcal* Enterotoxin B and tetrodotoxin. For other select toxins, the modeling results suggest a higher permissible limit. The output of this DSF analysis agrees with the current select toxin designations, with all select toxins, and none of the non-select toxins in Table 2 being recommended for inclusion. In general, setting a threshold of 10 exposures to an inhalation LD_50_ or TD_50_ and 100 exposures to an ingestion LD_50_ or TD_50_ in worse-case scenarios, resulted in recommended increases to previous exclusion limits for some of the select toxins (Table 4).

The DSF and Toxin Parameters and Attack Scenarios described in this paper are useful for the selection of toxins as select toxins and setting exclusion toxin amounts based on the best current information. During this study, we identified several gaps in our knowledge of the LD_50_ and TD_50_ values for inhalation and ingestion exposure. As we continue to fill these gaps and our knowledge evolves, this framework will allow decision makers to update the list of select toxins and associated exclusion toxin amounts in a consistent and data-based manner.

## Conclusion

The goal of this effort was to explore the use of DSF—logic tree approaches to identify toxins as select toxins. The output of this DSF analysis agrees with the current list of select toxins and did not identify any additional toxins for consideration at the current time. This approach was complemented with modeling studies of inhalation and ingestion scenarios to determine exclusion limits for scientists to possess and use without being subject to the Select Agent Regulation. Although we evaluated 30 toxins in this study, it is important to note that there are still scientific gaps associated with many of these toxins. As these gaps are addressed, the scientific community can continue to leverage this methodology to further refine this report’s findings in support of the Federal Select Agent Program. It is also important to note that based on the opinion of consulted SMEs, there is no need for any research laboratory to possess greater than 1 g of toxin to conduct any meaningful research to advance science. However, this is not a statutory criterion used to evaluate select toxins.

## Data Availability

The original contributions presented in the study are included in the article/supplementary material, further inquiries can be directed to the corresponding author.
